# Long-Term Structural Changes in the Osteochondral Unit in Patients with Osteoarthritis Undergoing Corrective Osteotomy with Platelet-Rich Plasma or Stromal Vascular Fraction Post-Treatment

**DOI:** 10.3390/biomedicines12051044

**Published:** 2024-05-09

**Authors:** Aleksey Prizov, Elena Tchetina, Aleksey Volkov, Ilya Eremin, Nikolay Zagorodniy, Fedor Lazko, Andrey Pulin, Evgeniy Belyak, Konstantin Kotenko, Gulnora Eshmotova, Svetlana Glukhova, Aleksandr Lila

**Affiliations:** 1 Department of Traumatology and Orthopaedics, RUDN University, Miklukho-Maklaya Str. 6, Moscow 117198, Russia; aprizov@yandex.ru (A.P.); zagorodniy51@mail.ru (N.Z.); fedor_lazko@mail.ru (F.L.); belyakevgen@mail.ru (E.B.); 2 Immunology and Molecular Biology Laboratory, Nasonova Research Institute of Rheumatology, Kashirskoe Shosse 34A, Moscow 115522, Russia; sveglukhova@yandex.ru (S.G.); amlila@mail.ru (A.L.); 3 Department of Pathological Anatomy, RUDN University, Miklukho-Maklaya Str. 6, Moscow 117198, Russia; alex.volkoff@gmail.com (A.V.); geshmotova@mail.ru (G.E.); 4 Laboratory of Bone Tissue Pathology, Research Institute of Human Morphology, n.a. akad A.P. Avtsyna, Petrovsky National Research Center of Surgery, Abrikosovsky lane 2, Moscow 119435, Russia; 5 Surgery Department, Petrovsky National Research Center of Surgery, Abrikosovsky lane 2, Moscow 119435, Russia; cd105@mail.ru (I.E.); direktor@med.ru (K.K.); 6 National Medical Research Center of Traumatology and Orthopedics, n.a. N.N. Priorov, Priorova Str. 10, Moscow 127299, Russia; 7 Pirogov National Medical and Surgical Center, Nizhnyaya Pervomayskaya Str. 70, Moscow 105203, Russia; andreypulin@gmail.com

**Keywords:** knee osteoarthritis, corrective osteotomy, platelet-rich plasma, stromal vascular fraction, osteochondral unit, morphometry

## Abstract

This pilot study examined the long-term structural changes in the osteochondral unit of 20 patients with knee osteoarthritis (KOA) who underwent high tibial osteotomy (HTO) and received post-treatment with either platelet-rich plasma (PRP) or stromal vascular fraction (SVF). Ten patients were injected with autologous PRP (PRP subgroup), while another ten patients received autologous SVF (SVF subgroup) six weeks after surgery and were monitored for 18 months. Histological samples of bone and cartilage (2 mm in diameter and 2 cm long) were taken from tibial and femoral sites during surgery and 18-month post-HTO, and morphometric analyses were conducted using Mega-Morf12 software. Both post-treatment resulted in an increase in articular cartilage height at both sites (*p* < 0.001 in the tibia and femur), indicating positive outcomes. Significant improvements in subchondral and trabecular bone architecture were also observed, with SVF injection showing higher reparative capacity in terms of bone volume (*p* < 0.001 for the tibia and *p* = 0.004 for the femur), subchondral bone height (*p* < 0.001 for the tibia and *p* = 0.014 for the femur), trabecular bone volume (*p* < 0.001 for the femur), and intertrabecular space (*p* = 0.009 for the tibia and *p* = 0.007 for the femur). This pilot study, for the first time, demonstrates that HTO surgery combined with PRP and SVF post-treatments can lead to significant enhancements in knee articular cartilage and bone architecture in KOA patients, with SVF showing higher regenerative potential. These findings may contribute to improving treatment strategies for better clinical outcomes in HTO therapy for patients with KOA.

## 1. Introduction

Knee osteoarthritis (KOA) is a systemic condition that affects the knee joints, involving degenerative changes in the articular cartilage, remodeling of the subchondral bone, and limited synovial inflammation associated with pain. While KOA is a whole joint disease involving all joint tissues, including articular cartilage, menisci, and infrapatellar fat pad, the disease impacts the entire osteochondral unit [1,2], including structural changes in the underlying subchondral and trabecular bones [3].

Articular cartilage characteristics are tailored for load transfer through a fibrillar collagen network primarily composed of type II collagen providing tensile strength and proteoglycan aggregates offering compressive resilience [4]. In OA progression, cartilage extracellular matrix degradation initiates in the superficial zone and extends to deeper regions [5], leading to fibrillations, microscopic cracks, fissures, delamination, and exposure of the calcified cartilage and subchondral bone [6].

The subchondral bone is located beneath the calcified cartilage and forms a structure similar to cortical bone [7]. Mathematical modeling studies predict that the subchondral bone serves to distribute loads and could protect overlying cartilage from damage [8], acting as a better shock absorber [9]. The subchondral bone combines the trabecular bone (or the cancellous bone), which is more porous and metabolically active than cortical bone [10]. The trabecular bone contains fatty bone marrow, and the trabeculae in the cancellous bone are oriented in different directions, providing a structural network adapted to local mechanical impacts [11].

During the development and progression of OA, changes in the subchondral bone, including decreased thickness, alterations in subchondral trabecular bone mass, and architecture [12], adversely affect the calcified and articular cartilage [13] and alter chondrocyte cellular function. Bone turnover disturbances alter tissue mineralization and change its susceptibility to structural damage [14].

In addition, knee OA often progresses due to incorrect alignment that affects load distribution at the joint, while varus deformity increases the risk of OA progression at the medial condyle [15] and worsens biomechanical gait parameters [16]. Osteotomy improves OA progression and symptoms by altering the mechanical environment of the joint [17]. It is a common and validated technique for painful medial compartmental OA of the knee with varus deformity, primarily for relatively young and active patients, producing good results with fewer drawbacks than other techniques in short- and mid-term follow-up [18]. In addition to the clinical improvement of the knee joint, determined by pain reduction, high tibial osteotomy (HTO) permits the restoration of normal gait biomechanics, as observed in our previous studies [19,20]. Furthermore, recent studies have demonstrated that HTO efficacy is increased when various post-treatments are applied [21]. However, there are currently only a few long-term prospective studies related to corrective osteotomy with post-treatments using biomaterials. These studies have shown better surgical results in terms of functional outcomes and pain scores [22,23,24,25]. At the same time, none of these studies have examined the morphology and morphometry of tissues from the whole osteochondral unit associated with HTO.

Recently, we demonstrated significant clinical and radiological improvements after HTO by incorporating additional procedures for cartilage regeneration, which involved adipose mesenchymal stem cells as part of the stromal vascular fraction (SVF) and platelet-rich plasma (PRP) injections into the knee [26]. The autologous SVF fraction is a heterogeneous group of cells enclosed within adipose tissue that is usually isolated by means of enzymes, such as collagenase [27]. PRP is the plasma fraction of whole blood, which has a platelet concentration above the baseline and contains major growth factors [21]. Our previous investigation in the same cohort of patients revealed that the PRP subgroup performed better in terms of KOOS, KSS, and VAS scores compared to the SVF subgroup. However, the SVF subgroup showed superior results in Outerbridge and Koshino testings, leading to more pronounced cartilage regeneration in the medial condyle while slowing down cartilage destruction in its lateral counterpart [26].

In this pilot study, we analyzed long-term structural changes in the entire osteochondral unit in the same cohort of patients with KOA undergoing corrective osteotomy with PRP or SVF post-treatment. To our knowledge, no studies have investigated the long-term effects of PRP or SVF post-treatment following HTO on bone and cartilage morphological and morphometric parameters.

## 2. Materials and Methods

Section 2.1. (Patients) and Section 2.2. (Surgical Techniques) are summarized, as they are exactly the same as in our previous study.

### 2.1. Patients

Twenty patients with knee OA (median age 54 years) and disease duration for 61.2 months were examined, which fulfilled the criteria of the American College of Rheumatology regarding OA [28]. This study followed the guidelines of the Declaration of Helsinki and was approved by the local ethical committee of Buyanov Moscow City Clinical Hospital (Protocol No. 06-07.04.17 on 7 April 2017). Written informed consent form was signed by all patients.

The inclusion criteria for this study were as follows: unrelated patients with knee OA aged between 20 and 65 years who visited Buyanov Moscow City Clinical Hospital from January 2020 to December 2021.

Exclusion criteria for this study encompassed patients with secondary osteoarthritis of the knee joint resulting from post-traumatic causes, septic arthritis, inflammatory joint diseases, gout, severe chondrocalcinosis, Paget’s disease, ochronosis, acromegaly, hemochromatosis, Wilson’s disease, primary osteochondromatosis, osteonecrosis, and hemophilia; chondromalacia of the articular cartilage of the lateral knee joint exceeding Grade 2 according to the Outerbridge classification [29], damage to the lateral meniscus beyond Stage 2 according to the Stoller classification [30], chronic concomitant somatic diseases in a stage of decompensation, restriction of knee joint extension ≥ 10°, systemic disease in their medical history, and a history of venous thromboembolism; substantial weight loss (>10%) of unknown origin in the previous year and the use of medications known to affect cartilage, bone, and adipose tissue metabolism; clinically significant abnormalities in laboratory test results; conditions that impede participation in this study and involvement in other clinical trials within 3 months prior to study commencement; patients with malignant tumors and Activated Partial Thromboplastin Time (APTT) increase > 1.8 times normal; a history of heterotopic ossification; and the prior use of glycoprotein IIB/IIIA inhibitors.

### 2.2. Surgical Techniques

The open wedge high tibial osteotomy procedure was carried out following established protocols and is described in detail in our previous study involving the same patient cohort [26]. Weight bearing on the operated joint was restricted for up to six weeks post-surgery. At the six-week mark, patients were divided into two subgroups: the PRP subgroup (10 patients: 5 men and 5 women) received knee injections of autologous PRP preparation (1.8 × 10^9^ cells per knee), while the SVF subgroup (10 subjects with knee OA: 5 men and 5 women) received injections of autologous SVF preparation (1.6 × 10^8^ cells per knee).

The concentration of platelets used in our study is in line with those published previously, as the dose of injected platelets varied from 0.21 to 5.43 × 10^9^ cells [31]. Cell numbers used in the SVF preparation were also in line with previously reported concentrations [32]. The subjects were divided into RPR and SVF subgroups according to the patient’s choice.

The detailed participants’ demographics for the examined patient subgroups are presented in Table 1. The patient subgroups did not differ significantly according to demographic data.

### 2.3. Preparation of Platelet-Rich Plasma (PRP)

Six weeks post-surgery, patients in the PRP subgroup had blood drawn from the cubital vein, obtaining a 40 mL sample for PRP preparation.

Subsequently, 2 mL of PRP was obtained through double centrifugation at 130× *g* for 15 min at room temperature (RT) to separate erythrocytes, followed by centrifugation at 250× *g* for 15 min at RT to concentrate platelets, as previously described [26,33]. A total of 2 mL of autologous PRP preparation was then injected into the patient’s knee joint.

### 2.4. Preparation of Stromal Vascular Fraction (SVF)

Six weeks following the surgery, patients in the SVF subgroup received local anesthesia. A team of plastic surgeons performed paraumbilical access to extract adipose tissue from the front of the abdominal wall using a syringe, collecting a volume ranging from 150 to 200 mL. The Celution 800/CRS device (Cytori Therapeutics Inc., San Diego, CA, USA) and enzymatic digestion with Cellase (Cytori Therapeutics Inc., San Diego, CA, USA) were employed to extract the SVF fraction from the autologous fat tissue, including cell counting and viability assessment, according to the manufacturer’s guidelines. Subsequently, 3.5 mL of the SVF preparation was injected into the patient’s knee joint [26].

### 2.5. Technique of Surgery and Sampling of Histological Material

**Figure 1:**
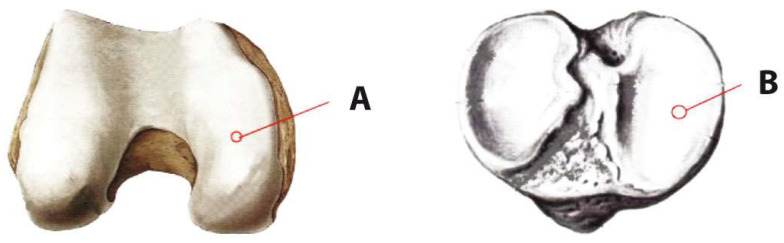
The site of tissue withdrawal in the case of the internal condyle of the femur (**A**) or tibia (**B**).

The surgery was performed under spinal anesthesia with the patient in the supine position. Arthroscopy was conducted on the most damaged articular surfaces of the femoral or tibial medial condyles. Histological material containing bone and cartilage (2 mm in diameter and 2 cm long) was extracted using an 11-gauge needle for bone biopsy (Figure 1). In the case of the medial femoral condyle, a standard medial arthroscopic approach was utilized with the knee flexed at 120 degrees. The needle was positioned in the central part of the internal condyle (in line with the cartilage defect) and was inserted to a depth of approximately 2 cm under arthroscopic guidance.

For biopsy retrieval from the tibial medial condyle, an additional upper medial access was employed. This access point was situated 2 cm proximal to the standard medial arthroscopic entry. An 11-gauge needle was inserted into the knee joint at maximum flexion, and it was advanced to a depth of around 2 cm using a hammer under arthroscopic visualization.

For a more detailed analysis of changes in bone and articular cartilage morphological characteristics, we conducted a histomorphometric examination of the osteochondral specimens obtained from patients with knee osteoarthritis who underwent corrective osteotomy with post-treatments using platelet-rich plasma (PRP) or stromal vascular fraction (SVF).

### 2.6. Histological Assay

Bone tissue specimens were fixed in 10% neutral formalin for 24 h followed by washing with running water. Standard paraffin histological processing was then performed using isopropyl alcohol (Biovitrum, St. Petersburg, Russia) at increasing concentrations. Isopropanol was substituted with paraffin (Biovitrum, St. Petersburg, Russia) with a threefold change. The tissue samples were embedded in blocks, from which 5 μm thick histological sections were cut. These sections were stained with hematoxylin and eosin (Biovitrum, St. Petersburg, Russia) and Mallory staining (Biovitrum, St. Petersburg, Russia). Photodocumentation was conducted using a digital camera microscope AxioLab A1 (Carl Zeiss AG, Oberkochen, Germany).

### 2.7. Morphometric Mesurements

Morphometric measurements of the bone tissues were performed using MegaMorf12 software (HistoLab, Moscow, Russia) to analyze linear, non-linear, and quantitative characteristics of the bone tissues following the manufacturer’s recommendations.

### 2.8. Statistical Analysis

Statistical analyses were conducted using Statistica software version 10.0 (StatSoft Inc., Tulsa, OK, USA). The Mann–Whitney U test was utilized to compare differences between subgroups, while the Wilcoxon signed-rank test was employed within subgroup analyses. Quantitative data were presented as medians [IQR, 25th; 75th percentiles]. Power analysis was performed using POWER analysis as a part of Statistica version 10.0 (StatSoft Inc., Tulsa, OK, USA). A *p*-value of ≤0.05 was deemed statistically significant. Significant differences are denoted by an asterisk (*).

## 3. Results

### 3.1. Clinical Parameters in the Examined Subjects with OA before and after Surgery with PRP or SVF Post-Treatment

The detailed clinical characteristics of the examined subgroups of patients with KOA have been presented in our previous paper involving the same subjects [26].

### 3.2. Comparison of the Histomorphometric Characteristics of the Examined Subgroups of Patients with OA Prior to Surgery

The intergroup analyses conducted before surgery revealed no differences in bone volume (BV) and subchondral (cortical) bone volume (Cr.V) in the medial condyles of the femur and tibia (*p* > 0.05) between patients with KOA treated with PRP (n = 10) or SVF (n = 10) after HTO surgery (Table 1 and Table S1). However, trabecular bone volume (Tr.V) was significantly higher in the tibia of SVF-treated patients but lower in the femur of the same patients compared with the PRP subgroup before surgery. Additionally, significantly higher subchondral (cortical) plate height (Cr.Wi) was noted in the medial condyles of the femur and tibia in PRP-treated patients compared with SVF-treated subjects. Articular cartilage thickness (Ch.Wi) was significantly higher in the medial tibial condyle of SVF-treated patients; however, the same subjects demonstrated lower cartilage thickness in the femoral medial condyles.

The results of an intergroup morphometric analysis of bone trabecular parameters, such as average trabecular thickness (Tr.Th) and average intratrabecular space (Tr.Sp), before HTO showed no significant differences in both subgroups at the baseline. The average number of trabeculae (Tr.N) before HTO was significantly higher in the medial tibial compartment in SVF-treated patients compared with the PRP subgroup (Table 2).

### 3.3. Morphological Description of Osteochondral Biopsies

#### 3.3.1. Morphology of Osteochondral Specimens before HTO in the SVF and PRP Subgroups of Patients with KOA

**Figure 2:**
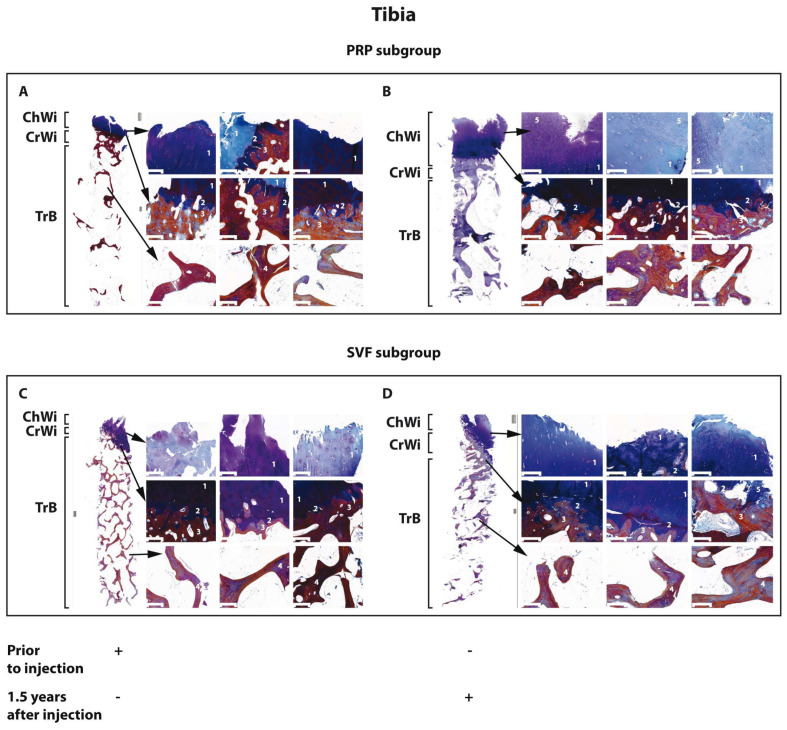
Morphology of osteochondral specimens from the tibia before (**A**,**C**) and 18 months after (**B**,**D**) HTO in the PRP (**A**,**B**) and SVF (**C**,**D**) subgroups of patients with KOA. Tr.B, trabecular bone; Cr.Wi, subchondral plate height; Ch.Wi, articular cartilage thickness; 1, hyaline cartilage; 2, mineralized cartilage; 3, subchondral bone plate; 4, bone trabecula; and 5, fibrous cartilage. Osteochondral specimen magnification level, 5×. Scale bar = 200 μm.

**Figure 3:**
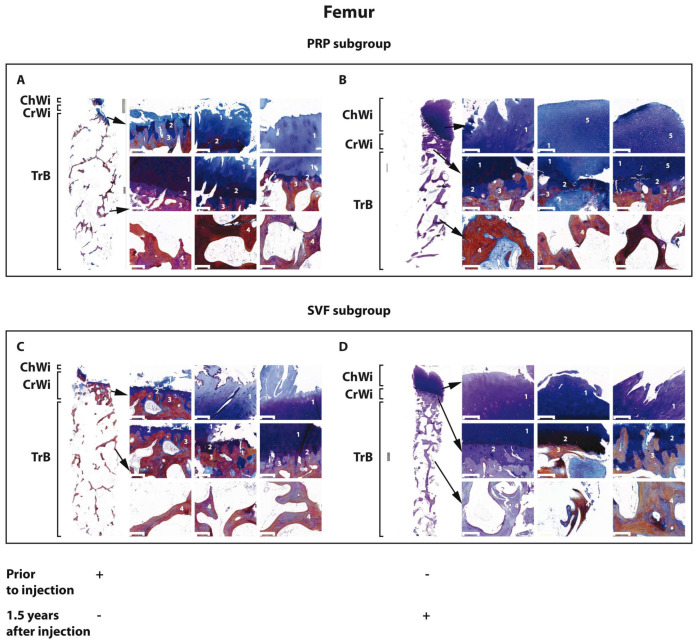
Morphology of osteochondral specimens from the femur before (**A**,**C**) and 18 months after (**B**,**D**) HTO in the PRP (**A**,**B**) and SVF (**C**,**D**) subgroups of patients with KOA. Tr.B, trabecular bone; Cr.Wi, subchondral plate height; Ch.Wi, articular cartilage thickness; 1, hyaline cartilage; 2, mineralized cartilage; 3, subchondral bone plate; 4, bone trabecula; and 5, fibrous cartilage. Osteochondral specimen magnification level, 5× Scale bar = 200 μm.

Histological examination of tissue specimens obtained from tibial and femoral sites revealed that the cartilaginous tissue (Ch.Wi) at the outer end of the specimen was either absent or had reduced height compared to healthy tissue (Figure 2 and Figure 3). Furthermore, hyaline cartilage was often replaced by fibrous tissue showing signs of degeneration. The subchondral bone (Cr.Wi) at the epiphysis displayed reduced height and exhibited trabecular formation activity. In cases where articular cartilage was completely absent, local osteosclerosis was observed. Trabeculae (Tr) of the cancellous bone were oriented longitudinally and perpendicularly to the axis and sometimes exhibited a honeycomb structure. The endosteum primarily consisted of resting cells, although in some samples, moderate osteoclastic activity was observed on the surface of cancellous bone trabeculae. The intertrabecular space was filled with yellow bone marrow.

#### 3.3.2. Morphology of Osteochondral Specimens 18 Months after HTO with the SVF and PRP Subgroups of Patients with KOA

At the end of the follow-up period, all examined specimens showed either hyaline cartilage or fibrous tissue at the outer end of the column (Ch.Wi). In the majority of samples, we observed dense fibrous connective tissue, primarily located in the subcortical zone, where osteoblastic activity was noted on the surface of bone trabeculae (Tr). We suggested that this accumulation of connective tissue might be a manifestation of adaptive remodeling (Figure 2 and Figure 3). In contrast to the pre-surgery state, trabeculae in the cancellous bone were aligned longitudinally relative to the articular surface. However, the honeycomb structure of the trabecular bone was also observed at times. The endosteum mainly consisted of resting cells. In most samples, osteoclastic activity was moderately expressed on the surface of cancellous bone trabeculae. Additionally, foci of osteogenesis and osteoclastic resorption were noted on the surface of some trabeculae and the subchondral bone, likely as part of remodeling activity. The intertrabecular space was filled with yellow bone marrow.

### 3.4. Comparison of the Histomorphometric Characteristics of the Examined Subgroups of Patients with OA Prior to Surgery and 18 Months after HTO Surgery with SVF or PRP Post-Treatment

#### 3.4.1. Assessment of the Relative Bone Volume at the Tibial and Femoral Sites in Patients with KOA before and 18 Months after HTO Surgery with SVF or PRP Post-Treatment

**Figure 4:**
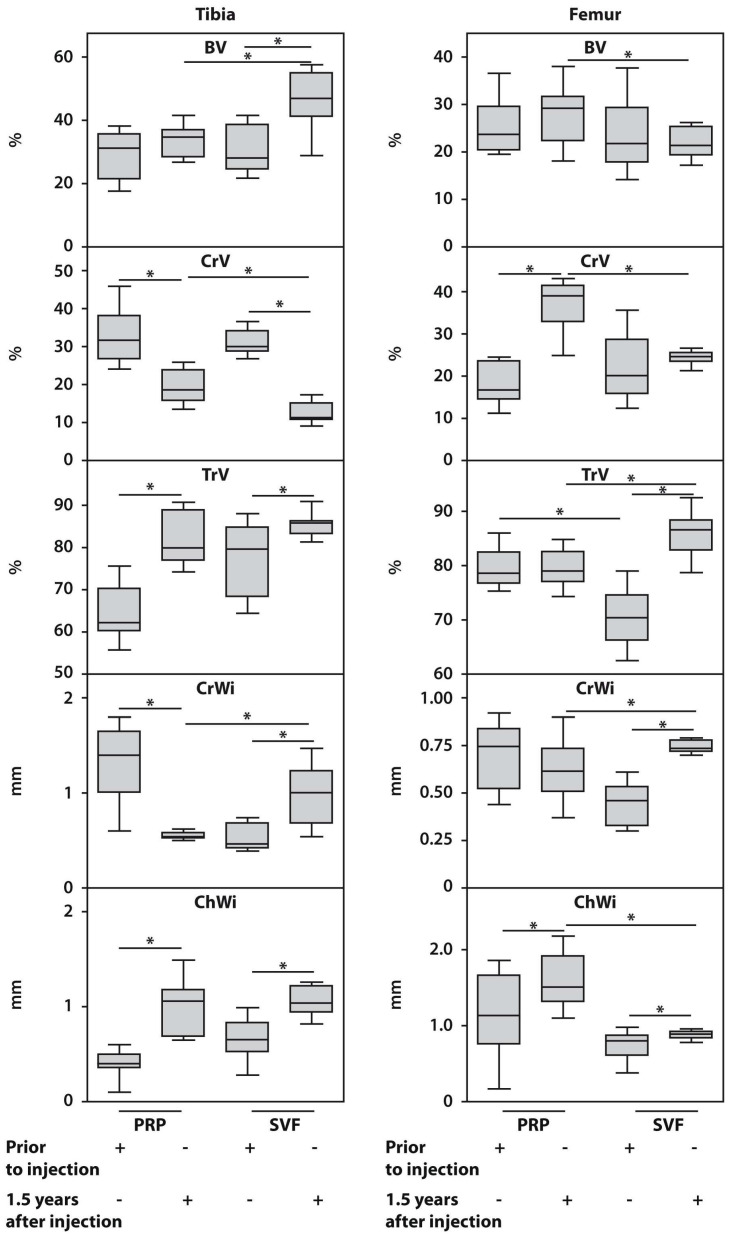
Histomorphometric characteristics of bone and articular cartilage at the tibial and femoral sites in SVF and PRP subgroups of patients with KOA before and 18 months after HTO surgery with SVF or PRP post-treatment. Asterisk (*) indicates significant differences (Mann–Whitney U test) between examined subsets. BV, bone volume; Cr.V, subchondral bone volume; Tr.V, trabecular bone volume; Cr.Wi, subchondral plate height; Ch.Wi, articular cartilage thickness.

The intragroup comparisons at 18 months post-surgery showed a significant increase in bone volume (BV) and trabecular bone volume (Tr.V) in the tibial medial condyles of the SVF subgroup compared to baseline levels along with a decrease in cortical bone volume (Cr.V) (*p* < 0.05) in the same subgroup. In contrast, for the femoral medial condyles, only an increase in trabecular bone volume (Tr.V) was observed at the end of the follow-up period, with no differences noted in bone volume (BV) and cortical bone volume (Cr.V) in the same patients compared to the baseline (Figure 4, Table S1).

In the intragroup comparisons of PRP-treated patients, a significant decrease in cortical bone volume (Cr.V) and an increase in trabecular bone volume (Tr.V) were observed at the end of the follow-up compared to the baseline in the medial tibial condyles. However, no differences were observed related to bone volume (BV). For femoral medial condyles, there was only a significant increase in trabecular bone volume (Tr.V) by the end of the follow-up compared to the baseline in patients in the PRP subgroup.

At the end of the follow-up period, the intergroup comparisons revealed significantly lower femoral bone volume (BV) and femoral cortical bone volume (Cr.V) in SVF-treated patients compared to PRP-treated subjects. In the SVF-treated subgroup, tibial cortical bone volume (Cr.V) was also lower compared to the PRP subgroup, but no differences were observed in tibial bone volume (BV) in both subgroups 18 months post-surgery.

#### 3.4.2. Assessments of the Relative Height of the Subchondral Bone and the Articular Cartilage Thickness in the Tibial and Femoral Sites before and after HTO Surgery with SVF or PRP Post-Treatment

The intragroup comparisons at 18 months post-surgery demonstrated a significant increase in subchondral bone height (Cr.Wi) and articular cartilage thickness (Ch.Wi) at the medial condyles of both the tibial and femoral sites in the SVF-treated group (Figure 4, Table S1). PRP-treated patients also showed an increase in articular cartilage thickness (Ch.Wi) at both the medial tibial and femoral condyles. However, a significant decrease in subchondral bone height (Cr.Wi) was observed at the medial tibial condyle in these patients, with no significant change noted in the medial femoral condyle.

At the end of the follow-up period, no significant differences were observed in subchondral bone height (Cr.Wi) and articular cartilage thickness (Ch.Wi) at the medial tibial condyles between the SVF and PRP subgroups. However, femoral subchondral bone height (Cr.Wi) was significantly higher in SVF-treated patients compared to the PRP subgroup, while articular cartilage thickness (Ch.Wi) was significantly lower at the femoral medial condyles in SVF-treated patients compared to the PRP subset after 18 months of follow-up.

#### 3.4.3. Assessment of the Tibial and Femoral Trabecular Bone Parameters before and after HTO in the SVF and PRP Subgroups of Patients with KOA

**Figure 5:**
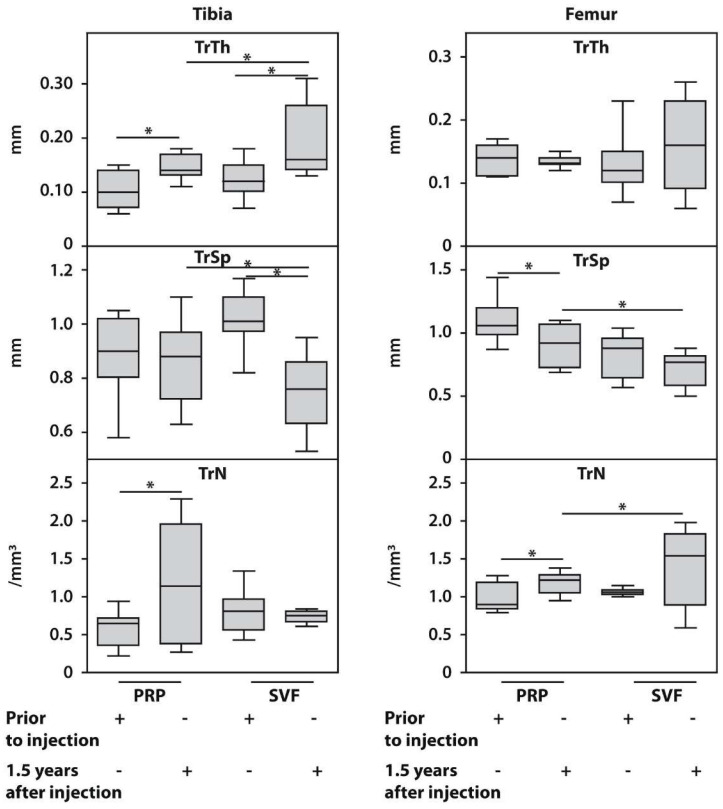
Histomorphometric characteristics of femoral and tibial trabecular bone parameters in the SVF and PRP subgroups of patients with KOA before and 18 months after HTO surgery with SVF or PRP post-treatment. Asterisk (*) indicates significant differences (Mann–Whitney U test) between examined subsets. Tr.Th, trabecular thickness; Tr.Sp, intertrabecular space; Tr.N, number of trabeculae.

The intragroup comparisons at 18 months post-surgery demonstrated that trabecular thickness (Tr.Th) significantly increased, while intertrabecular space (Tr.Sp) decreased (*p* < 0.05), both at the tibial and femoral medial condyles of patients treated with SVF compared with baseline scores. The trabecular numbers (Tr.N) were not different at the medial tibial condyle, while they significantly increased at the medial femoral condyle in the same subgroup compared with baseline scores (Figure 5, Table S1).

PRP-treated patients also showed a significant increase in trabecular thickness (Tr.Th) and trabecular numbers (Tr.N), as well as a decrease in intertrabecular space (Tr.Sp) at the medial tibial condyle 18 months after therapy compared with the baseline. However, at the medial femoral condyle, only trabecular numbers (Tr.N) increased by the end of follow-up compared with the baseline, while the other two parameters remained unchanged.

The intergroup morphometric analysis conducted after 18 months of follow-up demonstrated significantly higher trabecular thickness (Tr.Th) in the medial tibial condyle and lower intertrabecular space (Tr.Sp) (*p* < 0.01) in SVF-treated patients compared with those in the PRP subgroup. Additionally, trabecular numbers (Tr.N) at the medial femoral condyle in SVF-treated patients were also significantly higher compared with the PRP-treated subgroup at the end of follow-up. No other significant changes in the intergroup morphometric analysis of bone trabecular parameters were observed between the examined subgroups after 18 months of follow-up.

As we conducted a pilot study, we did not need to perform a priori sample size calculation. However, we conducted a post hoc power analysis of our results. The analysis revealed that this study was powered at 70% to 90% to detect a significant difference between the cartilage and bone parameters measured before both treatments and at the end of the follow-up period.

## 4. Discussion

The application of bone histomorphometric techniques has become a standard method for studying the osteochondral status and activities at the tissue level [34]. Understanding the articular cartilage and bone properties in terms of morphometric characteristics can provide profound insight into the pathogenesis of degenerative joint diseases [35]. However, studies on changes in osteochondral unit characteristics in response to HTO surgery with post-treatments have not been conducted before. In our previous study, we demonstrated that HTO surgery combined with PRP or SVF post-injection resulted in significant improvements in functional outcomes and pain scores [26]. With the present pilot study, we show that articular cartilage and bone morphometric characteristics were also affected. Moreover, the obtained results of morphometric evaluations are consistent with the data previously presented in other studies on histomorphometric characteristics of osteochondral units in patients with OA [15,36].

The progressive degeneration of articular cartilage is a fundamental issue in the pathogenesis of osteoarthritis, leading to a loss of joint function and often accompanied by severe pain. Therefore, inhibiting articular cartilage degeneration is a priority in the effective treatment of OA [37]. Indeed, we observed a significant increase in articular cartilage height after both PRP and SVF post-treatment compared with the baseline. Our findings are consistent with those of others. For example, two years after HTO, cartilage regeneration was observed at the medial tibial and femoral condyles in patients with medial knee OA [38]. Additionally, similar to other human and animal studies [39], we observed the formation of fibrocartilage covering bone areas and filling vertical clefts in hyaline cartilage. Furthermore, previously degenerated cartilage at the medial femoral condyle and the medial tibial plateau was shown to be covered by newly regenerated cartilage 1–2 years after HTO without chondrocyte implantation [40,41].

Cartilage serves to cover and protect the richly innervated subchondral bone from excessive stimulation, and under normal conditions, articular cartilage and subchondral bone interact in transmitting loading through joints [42]. In OA, the narrow, immature collagen fibers and decreased mineralization contribute to the decreased mechanical properties of the subchondral bone [43]. Furthermore, the anatomical region with cartilage pathology corresponds with changes in bone tissue, as stiffening of the subchondral bone leads to a decrease in shock absorbency with cartilage damage due to overload [44]. Therefore, in early-stage OA, the subchondral plate becomes thinner as a consequence of an increased remodeling rate [8]. Consequently, the further significant decrease in subchondral bone height in the case of PRP post-treatment at the tibial site and the absence of any changes in this parameter at the femoral site by the end of follow-up in our study cannot be considered a positive outcome of treatment in the examined patients with KOA. In contrast, SVF post-injection resulted in an increase in subchondral bone height at both examined sites. Moreover, at the femoral site, subchondral bone width was significantly higher compared with that in the PRP subgroup, indicating the positive effect of SVF post-injection and the higher reparative capacity of the latter post-treatment.

In early OA, the cancellous bone becomes osteopenic as the trabecular plates become thinner and more rod like [8]. Therefore, a significant increase in trabecular bone volume in response to both post-treatments by the end of follow-up, which was even more pronounced in the case of SVF post-injection at the femoral part compared with that in the case of PRP post-treatment, indicates a positive response to both post-treatments that improve the status of trabecular bone in patients with KOA after HTO surgery. The higher final trabecular bone volume in the case of SVF post-treatment indicates its higher reparative capacity.

Measurement of individual trabecular parameters such as trabecular thickness, trabecular numbers, and intertrabecular space provides a more detailed description of the overall trabecular architecture than bone volume alone. In normal bones, increases in bone volume fraction have been positively associated with increases in trabecular numbers and an increase in trabecular connectivity with no change in trabecular thickness [34]. Patients with OA commonly have significantly lower trabecular thickness, decreased numbers of trabeculae, and increased trabecular separation (space), primarily in the medial part of the tibial condyle, compared with healthy subjects [15,34]. However, as understanding the trabecular microstructure at the proximal tibial plateau is important for the success of prosthetic fixation at the bone–implant interface [34], studies of trabecular parameters are of special significance.

In this regard, a significant increase in trabecular thickness at the tibial condyle after both post-treatments might represent a positive outcome of these therapeutic approaches. Additionally, as patients with OA commonly have increased intertrabecular space compared with healthy subjects [15,45], a significant decrease in intertrabecular space in response to SVF post-treatment at the tibial medial condyle and after PRP post-injection at the femoral site could be considered a positive result of the corresponding treatments in the examined patients with KOA. Moreover, a significantly lower intertrabecular space at the femoral site in response to SVF post-treatment indicates the higher efficacy of this post-treatment compared with PRP post-injection.

As patients with OA commonly have decreased numbers of trabeculae compared with healthy subjects [15,45], a significant increase in trabecular numbers after PRP post-injection (at the tibial and femoral sites) and a significant accrue of trabecular numbers at the femoral site in response to SVF post-treatment could be considered a positive outcome of these treatments in the examined patients with KOA as they improve the trabecular bone architecture.

Bone volume (bone present per unit volume of a specimen) is the most common histomorphometric parameter measured as it is correlated with biomechanical properties, such as bone stiffness and strength [46]. Our results demonstrated no changes in bone volume at the tibial and femoral medial condyles in PRP-treated patients with KOA, indicating no improvement opportunity of this post-treatment related to bone volume. However, SVF post-treatment significantly increased bone volume in the medial tibial condyle, indicating a positive effect of SVF, as bone volume is usually decreased in patients with OA [8].

Our pilot study has some limitations related to the study design and small sample size. These issues limited our ability to carry out a perfect patient randomization process and calculate the sample size accurately.

Overall, in this study, we observed a higher reparative capacity of SVF post-treatment compared to PRP post-injection. This was evidenced by an increase in subchondral bone width, trabecular bone volume, trabecular numbers, and lower intertrabecular space. These results indicate a higher regenerative potential of SVF post-treatment.

Our findings are consistent with previous clinical results within the examined subgroups. The SVF subgroup showed better outcomes according to Outerbridge and Koshino testing, with more pronounced cartilage regeneration in the medial condyle and slowed-down cartilage destruction in its lateral counterpart. Additionally, the SVF subgroup exhibited a significant decrease in synovial proinflammatory cytokine concentrations, while concentrations of tissue regeneration-related fibroblast growth factor (FGF)2 increased [26]. FGF2 is known to be involved in angiogenesis, mesenchymal cell mitogenesis, and promoting the regeneration of articular cartilage [47,48].

Conversely, the PRP subgroup showed better results in femoral subchondral bone volume and articular cartilage thickness. It also performed better than the SVF subgroup in terms of physical function and pain scores [26]. This difference may be attributed to the increased amounts of systemic platelet-derived growth factor (PDGF)-AB/BB released during platelet aggregation. PDGF is capable of stimulating bone remodeling, extracellular matrix production, and revascularization [49].

## 5. Conclusions

In summary, we conducted, for the first time, a pilot study on long-term structural changes in the entire osteochondral unit in patients with knee osteoarthritis undergoing corrective osteotomy with PRP or SVF post-treatment. Our findings demonstrate that the degree of correction achieved with HTO surgery, in combination with PRP or SVF post-injection, led to significant improvements in knee articular cartilage and bone architecture in patients with KOA. The increase in articular cartilage height following HTO surgery, combined with PRP or SVF post-treatment, was also associated with positive changes in subchondral and trabecular bone architecture.

Furthermore, SVF post-injection showed a higher reparative capacity in relation to bone volume, subchondral bone height, trabecular bone volume, and trabeculae parameters, such as intertrabecular space, compared to PRP post-treatment. To further validate our results, additional studies involving larger cohorts of patients with KOA are needed. These future studies will help identify the optimal post-treatment combinations for corrective osteotomy surgeries.

## Figures and Tables

**Table 1:** Demographic characteristics of patients with KOA subjected to HTO therapy with post-surgery injections of PRP or SVF.

	PRP Subgroup (n = 10) Me [IQR]	SVF Subgroup (n = 10) Me [IQR]	*p* (Mann–Whitney U Test)
Age, years	56.5 [52.5; 63.5]	52.5 [45.0; 57.0]	0.089
BMI, kg/m^2^	30.2 [26.5; 33.2]	32.85 [25.25; 34.90]	0.393
Disease duration, months	21.0 [15.0; 78.0]	60.0 [27.0; 69.0]	0.424
Height, cm	165.0 [159.0; 177.0]	164.5 [163.0; 177.5]	0.795
Weight, kg	84.0 [76.0; 84.5]	90.0 [73.0; 103.0]	0.279

Note: PRP, platelet-rich plasma; SVF, stromal vascular fraction; Me [IQR], median [interquartile range, 25th, 75th percentiles].

**Table 2:** Histomorphometric characteristics of the examined subgroups of patients with KOA prior to surgery.

	PRP Subgroup (n = 10) Me [IQR]	SVF Subgroup (n = 10) Me [IQR]	*p* (Mann–Whitney U Test)
BV. %			
Tibia	31.2 [21.3; 35.7]	28.1 [24.4; 38.7]	*p* = 0.595
Femur	23.7 [20.3; 29.6]	21.8 [17.7; 29.4]	*p* = 0.305
Cr.V. %			
Tibia	31.7 [26.6; 38.2]	30 [28.6; 34.2]	*p* = 0.389
Femur	16.7 [14.4; 23.6]	20.1 [15.7; 28.7]	*p* = 0.116
Tr.V. %			
Tibia	62.2 [60.1; 70.3]	79.6 [68.2; 84.8]	*p* < 0.001
Femur	78.6 [76.6; 82.5]	70.4 [66.1; 74.6]	*p* < 0.001
Cr.Wi. mm			
Tibia	1.4 [1; 1.6]	0.47 [0.42; 0.68]	*p* < 0.001
Femur	0.75 [0.54; 0.84]	0.46 [0.33; 0.52]	*p* < 0.001
Ch.Wi mm			
Tibia	0.4 [0.4; 0.5]	0.66 [0.52; 0.83]	*p* < 0.001
Femur	1.14 [0.95; 1.64]	0.8 [0.61; 0.86]	*p* = 0.004
Tr.Th. μm			
Tibia	0.1 [0.07; 0.14]	0.12 [0.1; 0.15]	*p* = 0.249
Femur	0.14 [0.11; 0.16]	0.12 [0.1; 0.15]	*p* = 0.161
Tr.Sp. μm			
Tibia	0.9 [0.8; 1.02]	0.88 [0.64; 0.96]	*p* = 0.217
Femur	1.06 [0.98; 1.2]	1.01 [0.97; 1.1]	*p* = 0.161
Tr.N. n/mm^3^			
Tibia	0.65 [0.35; 0.72]	0.81 [0.55; 0.97]	*p* = 0.020
Femur	0.9 [0.83; 1.19]	1.06 [1.02; 1.09]	*p* = 0.202

Note: BV, bone volume; Cr.V, subchondral bone volume; Tr.V, trabecular bone volume; Cr.Wi, subchondral plate height; Ch.Wi, articular cartilage thickness; Tr.Th, trabecular thickness; Tr.Sp, intertrabecular space; Tr.N, number of trabeculae; PRP, platelet-rich plasma; SVF, stromal vascular fraction; Me [IQR], median [interquartile range, 25th, 75th percentiles].

## Data Availability

The data presented in this study are available upon request from the corresponding author.

## Supplementary Materials

The following supporting information can be downloaded at https://www.mdpi.com/article/10.3390/biomedicines12051044/s1, Table S1: Histomorphometric characteristics of the examined subgroups of patients with KOA prior to and 18 months after the injection.
