# Supplementary material for: Long-Term Structural Changes in the Osteochondral Unit in Patients with Osteoarthritis Undergoing Corrective Osteotomy with Platelet-Rich Plasma or Stromal Vascular Fraction Post-Treatment
Source: Biomedicines. 2024 May 9;12(5):1044. doi: 10.3390/biomedicines12051044 (PMC11118028; doi:10.3390/biomedicines12051044)
Supplement: Supplementary file 1 [file biomedicines-12-01044-s001.zip › biomedicines-2960862-supplementary.pdf]

Table S1. Histomorphometric characteristics of the examined subgroups of patients with KOA prior to and 18 months after the injection.

	PRP subgroup		SVF subgroup		p (Mann-Whitney U-test)
	Prior to injection	18 months after injection	Prior to injection	18 months after injection	
BV. %					
Tibia	31.2 [21.3;35.7]	34.7 [28.2;37.1]<br>**p=0.099[1]**	28.1 [24.4;38.7]	46.9<br>[41;55]<br>**p=0.001[1]***	p=0.595[2]<br>**p<0.001[3]***
Femur	23.7 [20.3;29.6]	29.2 [22.2;31.7]<br>p[1]=0.271	21.8 [17.7;29.4]	21.4 [19.2;25.4]<br>p[1]=0.532	p=0.305[2]<br>**p=0.004[3]***
Cr.V. %					
Tibia	31.7 [26.6;38.2]	18.6 [15.6;23.9]<br>**p<0.001[1]***	30 [28.6;34.2]	11.3 [10.6;15.2]<br>**p[1]<0.001***	p=0.389[2]<br>**p<0.001[3]***
Femur	16.7<br>[14.4; 23.6]	39 [32.7;41.5]<br>**p<0.001[1]***	20.1 [15.7;28.7]	24.6 [23.3;25.6]<br>p[1]=0.211	p=0.116[2]<br>**p<0.001[3]***
Tr.V. %					
Tibia	62.2 [60.1;70.3]	79.9 [76.8;88.9]<br>**p<0.001[1]***	79.6 [68.2;84.8]	85.8 [83.1;86.3]<br>**p=0.008[1]***	**p<0.001[2]***<br>p=0.148[3]
Femur	78.6 [76.6;82.5]	79 [76.9;82.6]<br>p[1]=0.865	70.4 [66.1;74.6]	86.6 [82.7;88.4]<br>**p<0.001[1]***	**p<0.001[2]***<br>**p<0.001[3]***
Cr.Wi. mm					
Tibia	1.4<br>[1;1.6]	0.54 [0.52;0.59]<br>**p<0.001[1]***	0.47 [0.42;0.68]	1<br>[0.68;1.24]<br>**p=0.001[1]***	**p<0.001[2]***<br>**p<0.001[3]***
Femur	0.75 [0.54;0.84]	0.62 [0.51;0.74]<br>p=0.244[1]	0.46 [0.33;0.52]	0.74 [0.72;0.78]<br>**p<0.001[1]***	**p<0.001[2]***<br>**p=0.014[3]**
Ch.Wi mm					
Tibia	0.4 [0.4;0.5]	1.06 [0.68;1.18]<br>**p<0.001[1]***	0.66 [0.52;0.83]	1.04 [0.94;1.22]<br>**p<0.001[1]***	**p=<0.001[2]***<br>p=0.436[3]
Femur	1.14 [0.95;1.64]	1.51 [1.31;1.92]<br>**p=0.019[1]***	0.8 [0.61;0.86]	0.89 [0.83;0.93]<br>**p=0.009[1]***	p=**0.004[2]***<br>**p<0.001[3]***
Tr.Th. μm					
Tibia	0.1 [0.07;0.14]	0.14<br>[0.13;0.17]<br>**p=0.006[1]***	0.12 [0.1;0.15]	0.16 [0.14;0.26]<br>**p=0.001[1]***	p=0.249[2]<br>**p=0.021[3]***
Femur	0.14 [0.11;0.16]	0.13 [0.13;0.14]<br>p=0.293[1]	0.12 [0.1;0.15]	0.16 [0.09;0.23]<br>**p=0.051[1]***	p=0.161[2]<br>p=0.126[3]

Tr.Sp. µm					
Tibia	1.06 [0.98;1.2]	0.92 [0.72;1.07]<br>**p=0.002[1]***	1.01 [0.97;1.1]	0.76 [0.63;0.86]<br>**p<0.001[1]***	p=0.217[2]<br>**p=0.009[3]***
Femur	0.9 [0.8;1.02]	0.88 [0.72;0.97]<br>p=0.177[1]	0.88 [0.64;0.96]	0.77 [0.58;0.82]<br>p=0.125[1]	p=0.161[2]<br>**p=0.007[3]***
Tr.N. n/mm^3					
Tibia	0.65 [0.35;0.72]	1.14 [0.37;1.96]<br>**p=0.009[1]***	0.81 [0.55;0.97]	0.75 [0.66;0.81]<br>p=0.532[1]	**p=0.026[2]***<br>p=0.148[3]
Femur	0.9 [0.83;1.19]	1.22 [1.04;1.29]<br>**p=0.017[1]***	1.06 [1.02;1.09]	1.54 [0.88;1.83]<br>**p=0.008[1]***	p=0.202[2]<br>**p=0.029[3]***

Note: All statistically significant differences are indicated with an asterisk (*)

[1] - Differences between values before and 18 months after the injection;

[2] – Differences between subgroups before injection

[3] - Differences between subgroups after the injection

BV, bone volume; Cr.V, subchondral bone volume; Tr.V, trabeculat bone volume; Cr.Wi, subchondral plate height; Ch.Wi, articular cartilage thickness. Tr.Th, trabecular thickness; Tr.Sp, intertrabecular space; Tr.N, number of trabeculae.
